# Unlocking the Mystery of the Therapeutic Effects of Chinese Medicine on Cancer

**DOI:** 10.3389/fphar.2020.601785

**Published:** 2021-01-15

**Authors:** Shao-Hsiang Liu, Po-Sheng Chen, Chun-Chieh Huang, Yi-Tu Hung, Mei-Ying Lee, Wei-Hung Lin, Yuan-Chuan Lin, Alan Yueh-Luen Lee

**Affiliations:** ^1^ Celgen Biotech, Taipei, Taiwan; ^2^ Taiwan Instrument Research Institute, National Applied Research Laboratories, Zhubei, Taiwan; ^3^ Jing-Her Chinese Medicine Clinic, Taipei, Taiwan; ^4^ Department of Chinese Medicine, Taitung Christian Hospital, Taitung, Taiwan; ^5^ HanPoo Chinese Medical Clinic, Taipei, Taiwan; ^6^ Chinese Medicine Women Doctors Association, Taipei, Taiwan; ^7^ Central Clinic & Hospital, Taipei, Taiwan; ^8^ Taipei Chinese Medical Association, Taipei, Taiwan; ^9^ National Institute of Cancer Research, National Health Research Institutes, Miaoli, Taiwan; ^10^ Graduate Institute of Biomedical Sciences, China Medical University, Taichung, Taiwan

**Keywords:** traditional Chinese medicine, cancer treatment, holistic approach, tumor microenvironment, cancer immunotherapy

## Abstract

Over the past decade, the rise of cancer immunotherapy has coincided with a remarkable breakthrough in cancer therapy, which attracted increased interests in public. The scientific community clearly showed that the emergence of immunotherapy is an inevitable outcome of a holistic approach for cancer treatment. It is well established that traditional Chinese medicine (TCM) utilizes the principle of homeostasis and balance to adjust the healthy status of body. TCM treatment toward cancer has a long history, and the diagnosis and treatment of tumors were discussed in the ancient and classical literatures of Chinese medicine, such as the Yellow Emperor’s Inner Canon. Precious heritage has laid the foundation for the innovation and development of cancer treatment with TCM. The modern study indicated that TCM facilitates the treatment of cancer and enhances the survival rate and life expectancy of patients. However, the pharmacological mechanisms underlying these effects are not yet completely understood. In addition, physicians cannot always explain why the TCM treatment is effective and the mechanism of action cannot be explained in scientific terms. Here, we attempted to provide insights into the development of TCM in the treatment and interpret how TCM practitioners treat cancer through six general principles of TCM by using modern scientific language and terms based on newly discovered evidence.

## Introduction

Cancer is a complex and confusing disease. As the number of in-depth studies increases, cancer becomes more difficult to classify and understand for therapy (Hoadley et al., 2018). Although many new drugs targeting cancer have been developed, researchers are unable to keep pace with variable and highly-evolving cancer (Gatenby, 2009; Hanahan and Weinberg, 2011; Willyard, 2016; McGranahan and Swanton, 2017). For instance, variations in the tumor microenvironment (TME), the changes in tumor metabolism, and the development of immunoescape in TME have become unsolved topics that impede us to deal with caancer (Vander Heiden and DeBerardinis, 2017; Jiménez-Sánchez et al., 2017; Zhuang et al., 2019).

In the past decade, there are enormous changes and revolutionary progressions occur in the area of cancer treatment, including the rise of cancer immunotherapy (Wei et al., 2018). However, cancer immunotherapy alone showed a low response rate and is only suitable to a small proportion of patients (Allison et al., 2020). The combination of other therapies with immunotherapy has recently been proved to be effective for increasing the cancer cure rate (Sharma and Allison, 2015; Swart et al., 2016). In addition, the efficacy of cancer immunotherapy is determined by the characteristics of TME, which regulates inflammation and the immunosurveillance (Fukumura et al., 2018; Schaaf et al., 2018). Therefore, a strategy of reducing inflammation and increasing immunity enhances the synergistic effect on the combination immunotherapy (Chen and Mellman, 2017). It is well-known that traditional Chinese medicine (TCM) is a combination of personal medicine and combinatorial cancer therapy (Liu et al., 2015). In the past, TCM acts as an anxiliary role for cancer treatment, especially after chemotherapy or radiotherapy. Currently, the majority of patients with cancer only takes TCM as an alternative therapy to provide nutritional and psychological comfort rather than therapeutic purposes (Xu et al., 2013; Liu et al., 2015), although cancer has been treated with TCM for many years and recorded in ancient medical books.

### Explanations for the Mystery Surrounding Traditional Chinese Medicine

TCM is based on a holistic view and considers the body as a complex and integrated system; therefore, its treatment has no standardization. Instead, the physician adjusted the medication and prescriptions according to different pathogenesis of patients, which can be considered as a kind of personalized medical treatment. However, sometime it is hard to give an explanation that why different diseases are treated with the same prescription (異病同治 in Mandarin/Chinese), and the same disease is treated with different prescriptions (同病異治 in Mandarin/Chinese). In addition, the most troublesome issue is scientific diagnosis because the same patient can be diagnosed by different doctors who recommend different treatments and even treated by opposite strategies (Liu et al., 2015). Another TCM mystery is its terminology. It is not described with modern scientific language and is often defined using idioms that only a trained, disciplined professional can understand. Besides, physicians cannot always explain why the treatment is effective and the mechanism of action cannot be explained in scientific terms. This communication problem persists today.

TCM mystery is further exacerbated by the current domesticated herbal cultivation practice that often produces herbs with no bioactive components. In the past, herbs were grown in the natural environment suitable for the species of herbs to obtain all the natural nutrients required to produce the therapeutic biologically active molecules. Based on this information, variations in the effectiveness of herbs from different origins and pharmaceutical companies have been observed. TCM practitioners who simply measure the weight of herbs instead of precisely measuring active ingredients coupled with patients returning to their own decoctions of drugs have contributed to the lack of standardization and scientific evidence. However, if the treatment was not effective, TCM would have been eliminated long ago, yet it has survived and enjoyed a revival in the global integrated health system. The most recent version of International Classification of Diseases (ICD-11) approved by the World Health Organization (WHO), is the first authority to include a chapter on TCM (2019). It reflects the wide-ranging impact of TCM on a global scale (Cyranoski, 2018). Further studies are needed to determine whether the international understanding and acceptance of TCM will change the field of biomedicine (Qiu, 2007).

### Six Strategies for Cancer Therapy by Traditional Chinese Medicine

TCM states that people become sick because of the disequilibrium of immunity. Diseases, including cancer, are also caused by internal or external evils, or by the inability of the body to balance an internal or external stimulus (Nagi et al., 2014). It has a long history for that cancer is treated with TCM. The diagnosis and treatment of tumors were discussed in the ancient and classical literatures of Chinese medicine, such as the Yellow Emperor’s Inner Canon (黃帝內經), more than 2000 years ago. The major concepts are strengthening body resistance and eliminating pathogens and treating both the manifestation and root cause. Precious heritage has laid the foundation for the innovation and development of cancer treatment with TCM (Liu et al., 2015). Here, we attempt to provide insights into the development of TCM in oncology and interpret how TCM practitioners treat cancer through six general strategies of TCM by using modern scientific language and terms based on newly discovered evidence (Table 1).

**TABLE 1:** Traditional Chinese medicine (TCM) formula for cancer treatment.

Strategy	Name of formula	Composition	Active compound	Mechanism of action	References
FuZhengQuXie (扶正祛邪)	Sijunzi decoction (四君子湯)	*Panax ginseng* C. A. Mey. 人參 *Glycyrrhiza uralensis* fisch. 炙甘草. *Poria cocos* (schw.) wolf 茯苓 *Atractylodes macrocephala* koidz. 白朮	β-Glucan/polysaccharide	Activation of macrophage. Anti-oxidation. Activation of dendritic cells and macrophages	(Zhou et al., 2019) (Huang et al., 2017) (Driscoll et al., 2009; Camilli et al., 2018)
		*Ganoderma lucidum* (lingzhi or reishi) 靈芝 *Cordyceps sinensis* (berk.) sacc. 蟲草	β-Glucan/polysaccharide Cordycepin	Activation of NK cells and macrophages. Anti-inflammation	(Driscoll et al., 2009; Camilli et al., 2018) (Qin et al., 2019)
QingReJieDu (清熱解毒)	SanHuangXieXin decoction (三黃瀉心湯)	*Rheum palmatum,* L.大黃 *Coptis chinensis* franch 黃連. *Scutellaria baicalensis* Georgi黃芩	Berberine Pheophorbide Wogonin	Anti-inflammation. Anti-angiogenesis. Pro-apoptosis. Induction of apoptosis/anti-inflammation	(Cheng et al., 2008) (Wang et al., 2015c) (Chan et al., 2006) (Chen et al., 2013; Xiao et al., 2015)
	Huanglian jiedu decoction (黃連解毒湯)	*Coptis chinensis* Franch黃連 *Phellodendron chinense* C. K. Schneid.黃柏. *Scutellaria baicalensis* Georgi黃芩. *Gardenia jasminoides* J.Ellis 梔子	Berberine Pheophorbide Wogonin	Anti-inflammation. Pro-apoptosis. Induction of apoptosis/anti-inflammation. Anti-proliferation	(Huang et al., 2020) (Chan et al., 2006) (Chen et al., 2013; Xiao et al., 2015) (Wang et al., 2015b)
		Cruciferous vegetables	Sulforaphane Indole-3-carbinol	Anti-oxidation	(Traka et al., 2014) (Metidji et al., 2018)
HuoXueHuaYu (活血化瘀	Didang decoction (抵當湯	*Rheum palmatum,* L.大黃. *Prunus persica* (L.) batsch 桃仁. *Hirudo nipponica* whitman 水蛭. *Tabanus bivittatus* matsumura 虻蟲	Amygdalin Hirudin	Anti-inflammation anti-thrombosis	(Lin and Lin, 2011) (Wildenrath, 1995)
		*Salvia miltiorrhiza* bunge (danshen) 丹參. *Ophiocordyceps sinensis* 冬蟲夏草	Salvianic acid (danshensu) Cordycepin	Anti-inflammation anti-angiogenesis anti-thrombosis/vasodilatation. Anti-angiogenesis	(Zhang et al., 2010; Zhang et al., 2018) (Li et al., 2015) (Liu et al., 2020)
RuanJianSanJie (軟堅散結)	XiaoLuoWan (消瘰丸)	*Scrophularia ningpoensis* hemsl. 玄參. *Ostrea gigas* thunberg 牡蠣. *Fritillaria thunbergii* miq. 浙貝母. *Sargassum pallidum* 海藻	Calcium Verticine	Anti-proliferation. Anti-inflammation. Anti-nociception	(Chen et al., 2016b) (Xu et al., 2011; Wang et al., 2015a)
		*Carapax trionycis* (biejia) 鰲甲. *Trionyx sinensis* wiegmann. 中華鱉 bovine tracheal cartilage (BTC)	CT6	Inhibition of TGF-β1 and anti-fibrosis. Up-regulation of CREB-p and anti-fibrosis	(Hu et al., 2013; Hu et al., 2017) (Tan et al., 2006)^,^(Prudden, 1985)
HuaTanQuShi (化痰祛濕)	ErChen decoction (二陳湯)	*Pinelliae ternata* (thunb.) makino, 半夏. *Citrus reticulata* blanco 陳皮. *Poria cocos* (schw.) wolf 茯苓. *Glycyrrhiza uralensis* fisch. 炙甘草	Ephedrine, β-glucan Diosmin β-Glucan/polysaccharide	Phlegm-eliminating, anti-inflammatory, and anti-metabolic syndrome effects	(Ji et al., 2014; Xiu et al., 2015) (Vafa et al., 2019) (Driscoll et al., 2009; Camilli et al., 2018)
YangXinAnShen (養心安神)	Suanzaoren decoction (酸棗仁湯)	*Ziziphus jujuba* mill. Var. *spinosa* (bunge) 酸棗仁. *Poria cocos* (Schw) 茯苓. *Ligusticum chuanxiong* 川芎. *Glycyrrhiza uralensis* fisch. 甘草	Acidic polysaccharides Jujuboside A Chuanxiongzine/tetramethyl pyrazine	Activation of immune cells. Hypnotic Vasodilatation	(Zou et al., 2018) (Cao et al., 2010) (Xu et al., 2018)

TCM, traditional chinese medicine.

### FuZhengQuXie Strategy

The first principle is the FuZhengQuXie method (扶正祛邪 in Mandarin/Chinese), which is literally translated to strengthening the resistance of the body (vital qi) and supporting healthy energy to eliminate pathogenic factors (expel the evil). It plays a central role in the TCM treatment of disease and often resonates with Chinese medicine practitioners. “Zheng” is defined as a normal qi, which is a type of healthy energy. When this righteous force is insufficient, people will get sick (Leong et al., 2015).

In this method, TCM often uses the fungi *Ganoderma lucidum* (Lingzhi or Reishi), *Poria* (Fu-Ling, Mandarin/Chinese) and other species as drugs; the fungi contain β-glucan or polysaccharide, which is known to enhance the effect of the immune system (Driscoll et al., 2009). Unfortunately, only laboratory data are available, and evidence for the anti-cancer effects of mushrooms from clinical trials is lacking (Jiang et al., 2017). Recently, β-glucan-induced trained immunity was observed to reverse lipopolysaccharide-induced immune paralysis through metabolic reprogramming and maintaining the integrity of the tricarboxylic acid cycle (Domínguez-Andrés et al., 2019). Consistent with these findings, the metabolic rewiring of immune cells in the TME might enhance antitumor immunity (Li et al., 2019).

β-Glucan has been used as a potential cancer immunotherapy because it targets macrophages (Camilli et al., 2018; Cassetta and Kitamura, 2018; Domínguez-Andrés et al., 2019; Li et al., 2019) and NK cells (Chiba et al., 2014; Okabe and Medzhitov, 2014). Interestingly, β-glucan might restore NK cells in patients receiving treatment for cancer. β-Glucan also enhances hematopoietic recovery after bone marrow injury (Cramer et al., 2006) and modulates the expansion of myelopoiesis after chemotherapy (Mitroulis et al., 2018). The immune system should no longer only be viewed with the traditional concept of fighting pathogens, but also maintaining tissue and whole-body homeostasis (Rankin and Artis, 2018). β-Glucan induced trained immunity (innate immune memory) generated via pathogen recognition receptors (PRRs) may be the mechanism underlying the effects of FuZhengQuXie as a TCM (Rubartelli and Lotze, 2007; Nagi et al., 2014).

### QingReJieDu Strategy

The second principle is the QingReJieDu method (清熱解毒 in Mandarin/Chinese), which is defined as clearing away heat and toxic substances. The source of Heat (HuoQi) that leads to toxicity is considered to be oxidative stress and inflammation. It induces damage at the cellular level in various tissues and organs, resulting in cardiovascular disease, hepatic injury, autoimmunity, lung disease, disorders of the reproductive system, retinopathy, neurotoxicity, neurodegenerative diseases, and carcinogenesis (Kohen and Nyska, 2002).

The QingReJieDu method is one of cancer treatment principles in TCM that balances oxidative stress and detoxification, including the regulation of energy metabolism and reduction in the levels of harmful metabolites. Modulation of oxidative stress and redox signaling is critical to regulate the equilibrium of cancer stem cell state and metastasis (Luo et al., 2018). The tumor cells secrete inflammatory factors that promote the inflammation in the tumor microenvironment; inflammation also inhibits immune response in cancer patients (Coussens and Werb, 2002; Kuo et al., 2020). The chinese pactitioners used QingReJieDu method to treat cancer when there is “Heat evil” or “Heat toxin” in the process of pathological mechanism. There are two commonly used TCM prescriptions/formula, SanHuangXieXin decoction (三黃瀉心湯) and Huanglian Jiedu decoction (黃連解毒湯), which were used to achieve the heat-clearing and detoxifying treatment (Table 1). The formula named SanHuangXieXin decoction is made up of three herbs, which are *Coptis chinensis* Franch, *Scutellaria baicalensis* Georgi, and *Rheum palmatum* L. Both decoctions are comprised of *Coptidis chinensis* and *Scutellaria baicalensis.* Berberine in *Coptidis chinensis* can suppress inflammation in cancer (Cheng et al., 2008; Huang et al., 2020). Pheophorbide in *Scutellaria baicalensis* is able to induce apoptosis of tumor cells (Chan et al., 2006). Wogonin in *Scutellaria baicalensis* has anti-inflammatory activity and can induce apoptosis of tumor cells (Chen et al., 2013; Xiao et al., 2015). In addition, a previous report indicated that Huanglian Jiedu decoction targets eukaryotic elongation factor 2 to attenuate cancer progression of hepatocellular carcinoma (Wang et al., 2015b).

On the other hand, cauliflower and broccoli are considered by TCM to belong to a type of food known as GanHanYangYin (甘寒養陰 in Mandarin/Chinese). These vegetables gently cool the body and maintain the nutrition-based repair system. Based on the results of recent studies on cruciferous vegetables, their functions are attributed to a substance called sulforaphane (SFN). It increases the activity of the phase II enzyme system by promoting Nrf2 nuclear translocation to induce the expression of genes related to detoxification and the inhibition of oxidation stress (Traka et al., 2014). Additionally, Nrf2 regulates the expression of mitochondrial enzymes to restore cellular vitality (Holmström et al., 2016). In addition, indole-3-carbinol (I3C), a compound present in cruciferous vegetables as a natural aryl hydrocarbon receptor (AHR) ligand, prevents inflammatory damage by maintaining intestinal stem cell homeostasis and barrier integrity (Metidji et al., 2018). Novel findings also suggest the reactivation of PTEN tumor suppressor through I3C-mediated WWP1 inhibition as a potential strategy for cancer treatment (Lee and Chen, 2019). TCM herbal extracts containing polyphenolic compounds, such as ellagitannins (ETs) and ellagic acid (EA), which enhance the barrier integrity by regulating the levels of a microbial metabolite through the Nrf2 pathway (Ito, 2011; Singh et al., 2019), thus providing the clue of QingReJieDu.

### HuoXueHuaYu Strategy

The third principle is the HuoXueHuaYu method (活血化瘀 in Mandarin/Chinese), which is defined as invigorating blood circulation and eliminating stasis. Cancer cells, fibroblasts, immune cells, and vasculature endothelial cells consist of the tumor microenvironment (TME). Their interaction in the TME determines the property and nature of cancer tissues through secretion of cytokines and other substances throughout the environment, which is crucial to tumor progression and metastasis (Kuo et al., 2020). Cancer tissues in a microenvironment depend on angiogenesis to supply their need for nutrients and oxygen; vessel formation after angiogenesis also becomes a route to metastasis. However, the network of tumor-associated blood vessels is chaotic and leaky, which increases interstitial fluid (IFP) pressure and difficulty in transportation. The collapsed vessels result in tumor regions that are hypoxic; moreover, the glycolytic nature of the hypoxic tumor cell acidifies the pH in the TME. The hypoxic and acidic TME that facilitates the genetic and epigenetic alterations that enhance their inflammation and aggressiveness (Schaaf et al., 2018). This highly aberrant angiogenesis contributes to maintain the immunosuppressive TME and causes cancer cells to escape the immunosurveillance and resist immunotherapy (Fukumura et al., 2018). Thus a strategy of vascular normalization was used to reduce excess angiogenesis and increase immune cells infiltration that further enhance the synergistic effect on the combination immunotherapy (Huang et al., 2012; Sung et al., 2019).

In addition, the difficulty in vessel transportation contributes the cancer-platelet interaction, platelet activation, the coagulation, and thromboembolism, which protects cancer cells from shear stress and immunological attack and facilitates to form a new metastasis (Lu et al., 2019). From TCM’s point of view, the origin of tumorigenesis is mediated by TanYuQiZu (痰瘀氣阻 in Mandarin/Chinese). It is explained as the difficulty in vessel transportation causes the hypoxic and acidic TME (metabolic waste accumulation) to trigger the inflammation and immunosuppression (Chung et al., 2013; Jain, 2014), which is exacerbated by metabolic reprogramming into the glycolytic nature of cancer cells. It is a promising approach that if the treatment can inhibit extensive angiogenesis and make vascular normalization; at the same time, the treatment can allow for eliminating thrombosis and improve inflammatory TME, which promotes infiltration of immune cells and activates CD8^+^ T cells. TCM prescriptions containing pharmacological effects of components have an effect on cancers through anti-angiogenesis, anti-inflammation, and anti-thrombosis, and invigorating blood circulation. For example, amygdalin in Didang decoction (抵當湯) has a role in anti-inflammation, and both Salvianic acid (Danshensu) in *Salvia miltiorrhiza Bge.(*Danshen) and Cordycepin in *Cordyceps sinensis* have anti-inflammation and anti-angiogenesis activities (Zhang et al., 2010; Zhang et al., 2018; Liu et al., 2020). On the other hand, Hirudin in *Hirudo nipponica* (Didang decoction) has anti-thrombosis activitity (Wildenrath, 1995) (Table 1). Since cancer immunotherapy plays a major role on the stage of cancer therapy and several therapeutic approaches have targeted the hypoxic TME to improve the clinical outcome of antiangiogenic therapy (Rapisarda and Melillo, 2012; Sennino and McDonald, 2012), TCM will be expected to enhance vascular normalization as a strategy to increase immune cell infiltration by vessel and further enhance the combination immunotherapy.

### RuanJianSanJie Strategy

The fourth principle is the RuanJianSanJie method (軟堅散結 in Mandarin/Chinese), which means softening and resolving hard masses and dispersing the accumulation of a pathogenic texture. Based on clinical trials, TCM prescriptions containing extracellular matrix (ECM) components exert a curative effect on hyperplasia or tumors. Using this method, TCM practitioners used seaweed, *Carapax trionycis* (Biejia), and even exotic animal materials in the ancient period. Recently the TME and its cellular matrix were shown to play important roles in cancer treatment (Pearce et al., 2018; Tlsty and Gascard, 2019). This method could be translated to reorganize the ECM of the TME.

Currently, invasive tumor margins with high stromal TGFβ activity are recognized to be associated with tumors that do not contain T cells. TGFβ signaling has been a therapeutic target (Mariathasan et al., 2018; Tauriello et al., 2018). Galunisertib, a TGFβ inhibitor in development by Eli Lilly is in a phase II trial for the treatment of hepatocellular carcinoma (Harjes, 2018). *Carapax trionycis* (Biejia), a traditional Chinese medicine originating from the shell of *Trionyx sinensis* Wiegmann, was also used as a candidate treatment for liver fibrosis. Its active ingredients interfere with TGF-β1 signaling by decreasing Smad three phosphorylation in cultured hepatic stellate cells (HSC), which are the main ECM-producing cells (Hu et al., 2013).

Many studies have focused on cartilage components with anti-angiogenesis activity in cancer therapy in the past few years (Moses et al., 1990; Patra and Sandell, 2012; Foradori et al., 2014). Recently, sulfated hyaluronans (sHA), a component of the ECM, were reported to decrease TGF-β1 bioactivity in fibroblasts (Koehler et al., 2017). ECM components from animals, i.e., cartilage, with abundant glycosaminoglycans possess anti-angiogenesis activity and other functions. Coincidentally, the FDA-approved drug Catrix^®^ derived from bovine tracheal cartilage (BTC) has been used to heal wounds (Prudden et al., 1964) and treat cancer (Prudden et al., 1964), similar to *Carapax Trionycis* (Biejia) in TCM. Additional investigations of this type of crosstalk will reveal how ECM regulates the TME.

### HuaTanQuShi Strategy

The fifth principle is the HuaTanQuShi method (化痰祛濕 in Mandarin/Chinese), which is defined as eliminating water and waste through metabolism. Metabolic disease is a complex cluster of metabolic imbalance, including typically obesity, insulin resistance, dyslipidemia and hypertension, at increase risk of type 2 diabetes mellitus, atherosclerosis and cardiovascular events. Cancer is also a complex disease of metabolic imbalance or reprogramming (Hay, 2016). Many studies have shown an association between metabolic disease and inflammation that is associated with higher risk of incidence for cancer (Xing et al., 2015; Saltiel and Olefsky, 2017; Wang et al., 2019). The main pharmacological effects of WenDan decoction (溫膽湯) and ErChen decoction (二陳湯) on cancer may consist in the regulation of lipid and glucose metabolisms as well as immunomodulatory function (Chen et al., 2016a). Both formula are comprised of *Pinellia Ternata* and *Citrus reticulata*. The genus *Pinellia* had improved clinical therapeutic effects for eliminating phlegm, anti-inflammation, and lowering blood pressure, glucose and cholesterol levels (Ji et al., 2014; Xiu et al., 2015). Diosmin in *Citrus reticulata* has a role in phlegm-eliminating, anti-inflammatory, and anti-metabolic syndrome effects (Vafa et al., 2019) (Table 1).

### YangXinAnShen Strategy

The sixth principle is the YangXinAnShen method (養心安神 in Mandarin/Chinese), which is defined as tranquilizing mind. Many studies have shown an association between psychological stress and cancer progression and metastasis (Powell et al., 2013; Shin et al., 2016). For instance, depression is associated with higher risk of mortality for patients with cancer (Pinquart and Duberstein, 2010; Lin et al., 2018). It is reasonable that reducing pressure, tranquilizing mind, and relief of anxiety in cancer patients will help control tumor progression. It is well-established that adrenergic neurons largely contribute to tumor growth and progression (Magnon et al., 2013). An exciting study showed that carvedilol, an inhibitor of adrenergic signaling, will lighten a way to combat the tumor-driven formation of adrenergic neurons in head and neck cancer (Amit et al., 2020). A number of clinical trials have shown that patients with cancer benefit from TCM as an adjunct to conventional treatment (Liu et al., 2015; Liao et al., 2017). The fatigue and anxiety in patients with prostate cancer can be improved by the treatment of Kamikihitou (加味歸脾湯) by restoring the balance of the autonomic nervous system (Tamada et al., 2018). The retrospective cohort study showed that the treatment of Suanzaoren decoction (酸棗仁湯) (Cao et al., 2010) and TianWangBuXinDan (天王補心丹) improved overall mortality and survival in prostate cancer patients with depression (Lin et al., 2019) (Table 1).

## Conclusion and Perspectives

**FIGURE 1:**
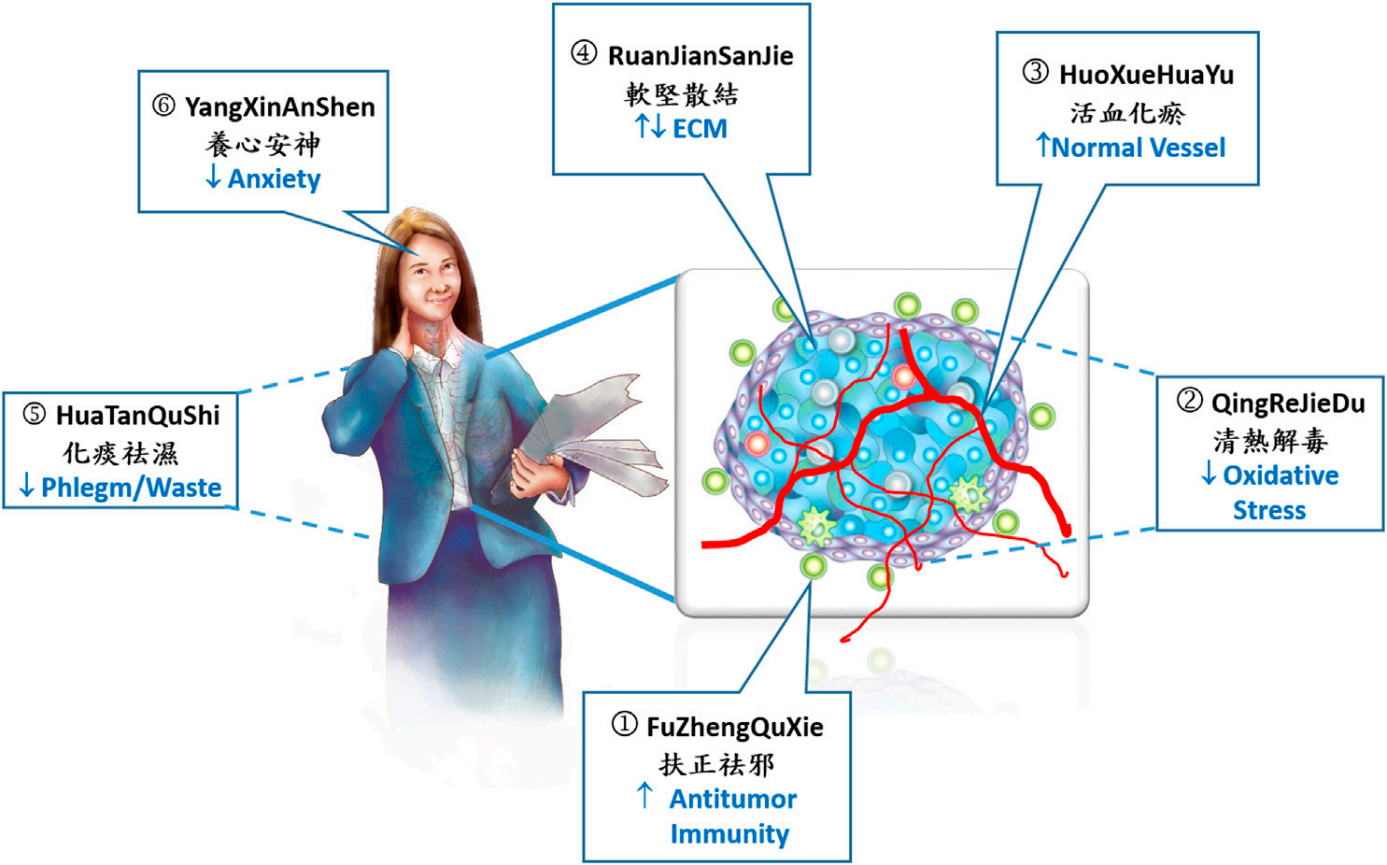
Six strategies for cancer therapy by TCM. Traditional Chinese medicine (TCM) utilizes six principles of homeostasis and balance to treat cancer. 1) Equilibrium of immunity plays a central role in the cancer treatment by TCM. 2) TCM balances detoxification and reduces oxidative stress and inflammation. 3) TCM utilizes a strategy of vascular normalization to reduce excess angiogenesis and increase immune cells infiltration that further enhance immunotherapy. 4) TCM regulates extracellular matrix (ECM) in the tumor microenvironment. 5) Restoration of metabolic imbalance and stimulation of phlegm-eliminating and anti-inflammatory activity by TCM. 6) Reduction of psychological stress and relief of anxiety by TCM decoction. In the tumor tissue: Blue irregular cells represent tumor cells. Green and blue circle cells represent infiltrated immune cells, T lymphocyte, neutrophil, and macrophage. Red line represents blood vessels. Purple cells represent stromal cells and ECM.

A better understanding of the principles of TCM based on the most recent evidence of scientific research will assist in the development of new cancer treatment. Here, we present the six major strategies for treating cancer used in TCM (Figure 1), and each strategy usually covers and targets various modern hallmarks of cancer (Hanahan and Weinberg, 2011), which reveals a combinatorial strategy of cancer therapy. This trend meets the concept of the tumor microenvironment and the rise of cancer immunotherapy, which represent a revolutionary change in the cancer treatment.

The main principle and philosophy of TCM is a holistic treatment, which is quite different from the Western medicine. Actually, TCM is also a combination of personal medicine and combinatorial cancer immunotherapy. After the diagnosis of inspecting, smelling, inquiring/listening, and taking palpation of the patient, Chinese medicine doctor then will distinguish the pattern of syndrome, sum up a pathological mechanism, and propose a prescription. To obtain an accurate prescription, Chinese medicine doctor may modify a traditional formulation by adding or subtracting herbs according to each individual patient. Of course, a TCM formulation is a kind of combination therapy. Therefore, the six strategies for cancer therapy mentioned in our work can be assortedly used in the treatment of cancer patients because cancer is a kind of complicated systematic disease. For example, the famous prescription of Xiao-Chai-Hu decoction (小柴胡湯) contains three strategies mentioned in the formulation for cancer treatment, including FuZhengQuXie, QingReJieDu, and HuaTanQuShi strategy. Xiao-Chai-Hu decoction contains *ginseng radix* and *Glycyrrhizae radix* that strengthen the resistance of the body (FuZhengQuXie), *scutellaria baicalensis* that clears away heat and toxic substances (QingReJieDu), and *Pinelliae ternata* that eliminates water and waste through metabolism (HuaTanQuShi). Accordingly, in recent years, researchers were starting to study TCM using the approach of system biology. They uncovered the interactive mechanisms of TCM formulas by using chemomic, transcriptomic, and/or proteomic approaches in relevant research (Lin et al., 2015; Chen et al., 2016a; Yin et al., 2020), which should be crucial for interpretation of TCM development. In the future, more studies on TCM formulas using systematic approaches are necessary to understand the interactive mechanisms of the formulas, which can be used directly in personalization medicine and the combination therapy of cancer patients with other therapies and immunotherapy in a holistic way.

In this review article, we have provided a new perspective knowledge of TCM in cancer therapy. Combined with the ancient wisdom of TCM and the language of modern science, we will look forward to finding new solutions to control cancer as a chronic disease with improved efficacy. Eventually, we hope that TCM is not just practical experiences, but a scientific medicine and holistic philosophy of therapy, which is quietly waiting and worthy for us to discover the truth and wisdom in the TCM.

## Data Availability Statement

The original contributions presented in the study are included in the article, further inquiries can be directed to the corresponding authors.

## Author Contributions

S-HL, P-SC, and AY-LL conceived the project. C-CH, Y-TH, M-YL, W-HL, and Y-CL provided discussion and suggestions to the manuscript. S-HL, P-SC, and AY-LL wrote the manuscript with input from all authors.

## Funding

This work was partly supported by grants from the Ministry of Science and Technology (MOST105-2628-B-400-003-MY3, MOST105-2627-M-400-002, MOST108-2320-B-400-008-MY3) and National Health Research Institutes (109A1-CA-PP-07), Taiwan to AY-LL.

## Conflict of Interest

S-HL is a chief technology officer of Celgen Biotech whose nutraceutical products contain β-glucan, sulforaphane, and bovine tracheal cartilage (BTC) components.

The remaining authors declare that the research was conducted in the absence of any commercial or financial relationships that could be construed as a potential conflict of interest.
